# Cysteamine suppresses human peripheral blood mononuclear cells – human corneal endothelial cell reaction via reactive oxygen species reduction

**Published:** 2011-12-21

**Authors:** Young Joo Shin, Joon Young Hyon, Seonhowa Kim, Jae Woong Koh, Soon Il Kwon, Won Ryang Wee

**Affiliations:** 1 Department of Ophthalmology, Hallym University College of Medicine, Seoul, Republic of Korea; 2 Department of Ophthalmology, Seoul National University College of Medicine, Seoul, Republic of Korea; 3 Department of Ophthalmology, Chosun University School of Medicine, Gwangju, Republic of Korea

## Abstract

**Purpose:**

To investigate the effect of cysteamine (CYS) on mixed peripheral blood mononuclear cells (PBMCs) - human corneal endothelial cell (HCEC) reaction (MLER).

**Methods:**

PBMC stimulation assay was performed using cultured HCEC. MLERs were treated with various concentrations of CYS (0–20 mM). The proliferation rate and secretion profiles of transforming growth factor-β1 (TGF-β1) and interleukin-6 (IL-6) of PBMCs stimulated by cultured HCEC were determined using bromodeoxyuridine proliferation assay and enzyme-linked immunosorbent assay, respectively.

**Results:**

CYS suppressed PBMC proliferation in a dose-dependent manner (p<0.001). The intracellular reactive oxygen species (ROS) levels decreased with an increase in CYS concentration (p<0.001). The levels of TGF-β1 and IL-6 decreased in a dose-dependent manner as well (p=0.011 and 0.003, respectively).

**Conclusions:**

This study showed that CYS decreased PBMC proliferation, IL-6 and TGF-β1 levels via ROS formation. Our results suggest that CYS could suppress inflammation associated with PBMCs to corneal endothelial cells.

## Introduction

Allograft corneal transplantation is widely performed to treat corneal diseases, including bullous keratopathy, Fuchs’ endothelial dystrophy, and corneal opacity [1]. Rejection is a major complication after allograft corneal transplantation [1-3]. The cornea has been described as an immune-privileged site because cornea has no blood or lymphatic vessels [4]. Allograft rejection after penetrating keratoplasty or endothelial transplantation. However, corneal endothelial rejection can cause persistent graft edema and failure [1,5] while epithelial rejection is usually self limited. Suppression of corneal endothelial rejection is important to maintain endothelial function and transparency of grafts [6]. Many drugs have been developed to suppress the inflammation associated with organ transplantation including corneal endothelial rejection after corneal transplant [7-9]. The mainstay is the use of immunosuppressive agents including rapamycin, steroid, and cyclosporine A [10].

Recently, reactive oxygen species (ROS) has been described to induce the inflammation [11,12]. It has been reported that antioxidants play an essential role in inhibiting inflammation [13,14]. However, there has been no study about the role of antioxidants in corneal endothelial rejection after corneal transplantation. Cysteamine (CYS) is a simple aminothiol that participates in various physiologic processes, including amino acid transport, disulfide reduction, protein synthesis, and protection against oxidative damage [15-18]. CYS has been described as an anti-oxidant [19]. CYS enhances glutathione (GSH) synthesis under various forms of toxic stress [18]; furthermore, it exerts a potent and prolonged anti-inflammatory effect [20,21]. In this study, we investigated the effects of CYS on corneal endothelilal rejection via the interactions between peripheral blood mononuclear cells (PBMCs) and human corneal endothelial cells (HCECs).

## Methods

This study was performed according to the tenets of the Declaration of Helsinki, and was reviewed and approved by the institutional review board/ethics committee of Hallym University Medical Center. HCECs were obtained from discarded corneal-scleral rings after penetrating keratoplasty. These tissues were stored in Optisol^™^-GS (Bausch and Lomb Inc., Rochester, NY) at 4 °C until processed for culture.

### Human corneal endothelial cell culture

HCECs were cultured in accordance with previously published methods [22]. HCECs from the remnant donor tissues after corneal transplantation were harvested on or before the 7th day after death. All of the cells remained attached to Descemet’s membrane. The endothelial cells and Descemet’s membrane complex were incubated for 1 h in 0.02% EDTA solution, stirred vigorously with a flame-polished pipette to disrupt cell junctions, centrifuged for 5 min at 3000× g, and seeded onto culture plates coated with FNC coating mix (Athena Enzyme System, Baltimore, MD) containing bovine fibronectin (10 μg/ml) and bovine type I collagen (35 μg/ml). The cells were then cultured in OptiMem-I media (GIBCO/BRL Life technologies, Grand Island, NY) supplemented with 8% FBS (Cambrex Bio Science, Walkersville, MD), 200 mg/l of calcium chloride (Sigma Chemical Co. St. Louis, MO), 0.08% chondroitin sulfate (Sigma Chemical Co.), 20 μg/ml ascorbic acid (Sigma Chemical Co.), 100 μg/ml pituitary extract (Invitrogen, Grand Island, NY), 5 ng/ml epidermal growth factor (Sigma Chemical Co.), 20 ng/ml nerve growth factor (Sigma Chemical Co.), 10 μg/ml gentamicin (Invitrogen), 100 IU/ml penicillin (Cambrex Bio Science, Walkersville, MD), 100 IU/ml streptomycin (Cambrex Bio Science), and 2.5 μg/ml amphotericin (Cambrex Bio Science) under an atmosphere of 5% CO_2_. The medium was changed every 2 days. At confluence, the cells were split 1 to 3, and cells from passage 4 were used for experiments.

### Immunofluorescence staining

HCECs cultured on cover glasses in 12-well plates were washed with phosphate buffered saline (PBS) and fixed for 20 min in 3.7% formaldehyde solution. The cells were permeabilized for 10 min with 0.5% Triton X-100 and blocked for 1 h with 1% BSA at room temperature. After washing, the cells were incubated overnight with rabbit polyclonal antibody to zonular occludence-1 (ZO-1; Zymed Laboratories, San Francisco, CA) at 4 °C, then washed with PBS. The cells were incubated with FITC-conjugated donkey anti-rabbit IgG antibody (1:100) for 1 h at 37 °C in the dark, then counterstained with Hoechst nuclear staining dye (1:2,000; Molecular Probes, Leiden, The Netherlands) in accordance with the manufacturer’s recommendations. After extensive washing with PBS, the slides were mounted in a drop of mounting medium to reduce photobleaching. Negative control staining was conducted in parallel with the omission of primary antibodies.

### PBMC isolation

Heparinized fresh whole blood (10 IU heparin/ml) was diluted 1:2 with PBS solution. The peripheral blood mononuclear cell (PBMC) fraction was obtained by Ficoll-Hypaque centrifugation. The cells were then washed in PBS before culture. The PBMCs were cultured for 24 h at 37 °C at a density of 1×10^6^ cells/well in Roswell Park Memorial Institute (RPMI) medium supplemented with 5% (vol/vol) fetal calf serum. The viability of PBMCs was measured by trypan blue dye exclusion and was consistently greater than 98%. The cells were then suspended in RPMI-1640 (Invitrogen-Life Technologies).

### Mixed PBMC–HCEC reaction (MLER)

PBMC stimulation assay was performed to determine immunoreactivity, as previously described [23,24]. In this study, mitomycin C-treated HCECs (5×10^5^/ml) were used as stimulating cells. These cells were incubated with 25 µg/ml mitomycin C for 30 min in a 5% CO_2_ humidified incubator. Residual mitomycin C was removed by repeated washing (3 times) with RPMI 1640 containing 10% fetal bovine serum. Then, the stimulating and responding cells were cocultured in 100 μl of RPMI 1640 in 96-well plates and incubated for 2 days either with 0–20 mM CYS. After incubation, PBMC proliferation was measured using a commercial bromodeoxyuridine (BrdU) proliferation assay kit (Roche Diagnostics GmbH., Mannheim, Germany) according to the manufacturer’s protocol. One hundred microliters of PBMC cell suspension (1×10^6^ cells/well) was added to 5 wells of flat-bottomed 96-well plates. Unstimulated PBMCs served as the negative control, whereas PBMCs treated with phytohemagglutinin (PHA, 5 µg/ml; Sigma Chemical Co.) served as the positive control. Optical density was measured at 450 nm with an enzyme-linked immunosorbent assay (ELISA) reader. The results are expressed as mean (standard deviation [SD]). Three times of experiments were repeated. Proliferation rates were calculated as the percentage increase in the number of cells between PHA-stimulated and unstimulated cultures after subtracting the corresponding blanks. Proliferation rates were calculated as follows: percentage of BrdU proliferation (%)=(mean optical density (O.D.) in sample – mean O.D. in unstimulated cultures) / (mean O.D. in PHA-stimulated cultures - mean O.D. in unstimulated cultures) × 100.

### Enzyme-linked immunosorbent assay (ELISA)

The supernatant was collected after centrifugation at 300× g for 10 min, and frozen at −70 °C until used to determine the cytokine levels. The levels of interleukin-6 (IL-6) and transforming growth factor-β (TGF-β) in the PBMC culture medium were assayed using commercial human IL-6 and TGF-β ELISA kits (R&D Systems, Minneapolis, MN) according to the manufacturer’s protocols. The sensitivity limit of each ELISA was ~15 pg/ml. In brief, anti-human TGF-β1 or IL-6 antibody was added to each well of a 96-well microtiter plate and incubated overnight at room temperature (RT). The next day, each well was washed with washing buffer, filled with a blocking buffer (pH 7.4) containing 1% BSA, and then incubated at RT for 1 h. One hundred-microliter standard dilutions of human TGF-β1 or IL-6 (obtained from the experimental samples) were dispensed into the wells of a 96-well microtiter plate; these wells were coated with an appropriate polyclonal antibody. The plate was sealed and incubated at RT for 2 h. After incubation, the plates were washed 4 times, and 100 μl of goat anti-human TGF-β1 or IL-6 antibody conjugated to horseradish peroxidase was added to each well, and the plates were incubated at RT for 2 h. One hundred-microliter aliquots of a color reagent (3,3′,5,5′-tetramethylbenzidine [TMB]) were then applied to the plates for 20 min to obtain a blue color; the reaction was stopped by adding 50 μl of 1 M H2SO4. The absorbance was measured at 450 nm by using an automatic plate reader with a 570 nm reference wavelength.

### Measurement of intracellular reactive oxygen species formation

Reactive oxygen species (ROS) formation was measured using the oxidation-sensitive fluorescent probe, 2′7′-dichlorofluorescein diacetate (DCFH-DA; D6665; Sigma Chemical Co.); this measurement is based on the ROS-dependent oxidation of DCFH-DA to 2′7′-dichlorofluorescein (DCF). MLERs were incubated with 0–20 mM CYS for 48 h, suspension cells were collected, and were then centrifuged at 300× g for 10 min. The supernatant was discarded, and the cells were washed with PBS. Then, the cell suspension was centrifuged at 300× g for 10 min. Subsequently, 200 µl of 10 μM DCFH-DA was added, and the cells were incubated for further 30 min at 37 °C in darkness. These cells were then washed with PBS. Intracellular ROS production was measured using the previously described DCFH-DA microplate assay method [22]. We used a spectrofluorometer (SFM 25; Kontron Instruments, Basel, Switzerland) to measure ROS generation by the fluorescence intensity in each well at an excitation wavelength of 495 nm and an emissions wavelength of 530 nm. Relative intensities of DCF fluorescence were calculated by setting the fluorescence intensity of the PHA-stimulated cultures to 100% after subtracting the corresponding blanks.

## Results

### HCECs culture

**Figure 1:**
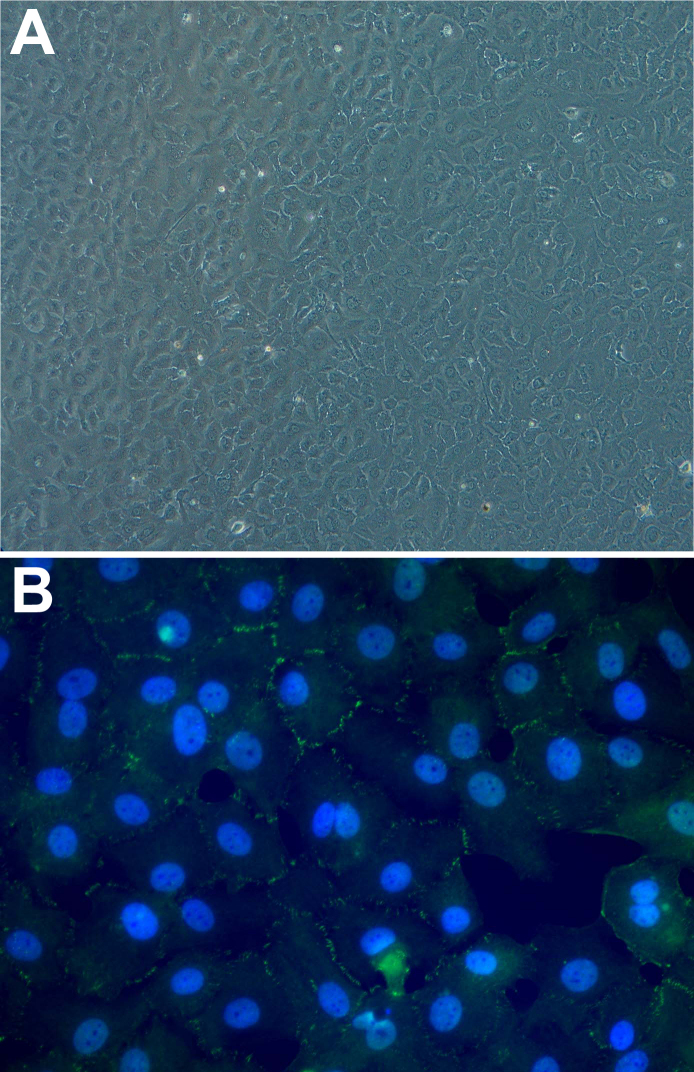
Human corneal endothelial cell examination. Inverted phase-contrast imaging (A) and immunofluorescent ZO-1 staining (B) of the human corneal endothelial cells. Human corneal endothelial cells were observed in mosaic pattern. Immunofluorescence with anti-ZO-1 antibody, a general marker of the tight junction, revealed the morphology. The nuclei were stained using Hoechst 33342 (blue). Magnification, 200×.

The cultured HCECs showed growth (Figure 1A). Immunofluorescence with ZO-1, a marker of the tight junction, revealed the morphology (Figure 1B). The nuclei were stained with Hoechst Mann–Whitney U test).

### Intracellular ROS measurement

**Figure 2:**
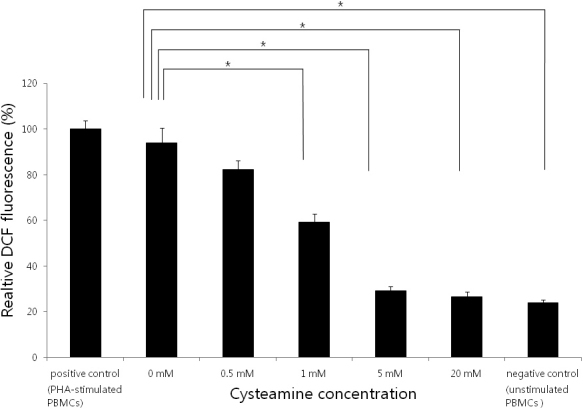
Relative 2′′dichlorofluorescein (DCF) fluorescence. CYS decreased intracellular ROS levels in a dose-dependent manner (p<0.001; Kruskal–Wallis test). PHA-stimulated PBMCs served as the positive control and unstimulated PBMCs served as the negative control. *: statistically significant by the Mann–Whitney U test.

CYS decreased intracellular ROS levels in a dose-dependent manner (p<0.001; Kruskal–Wallis test; Figure 2). The DCF assay showed that a CYS concentration of ≥0.5 mM reduced the intracellular ROS levels (p=0.006, 0.006, 0.004, and 0.004, respectively; Mann–Whitney U test).

### IL-6 secretion profiles of activated PBMCs

**Figure 3:**
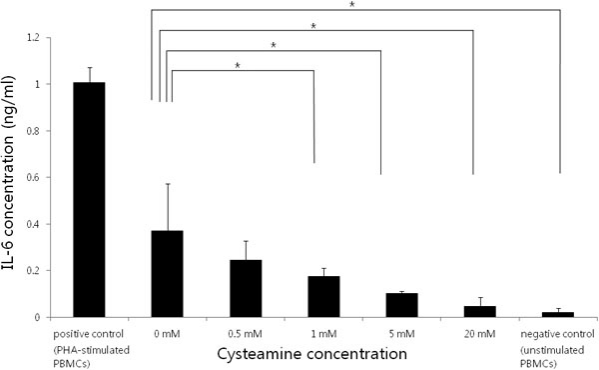
TGF-β levels measured by enzyme-linked immunosorbent assay (ELISA). CYS decreased TGF-β levels in a dose-dependent manner (p=0.011; Kruskal–Wallis test). PHA-stimulated PBMCs served as the positive control and unstimulated PBMCs served as the negative control. *: statistically significant by the Mann–Whitney U test.

CYS decreased IL-6 levels in a dose-dependent manner (p=0.003; Kruskal–Wallis test; Figure 3). CYS concentration of ≥1 mM significantly lowered the IL-6 levels (p=0.034, 0.021, and 0.021, respectively; Mann–Whitney U test).

### TGF-β1 secretion profiles of activated PBMCs

**Figure 4:**
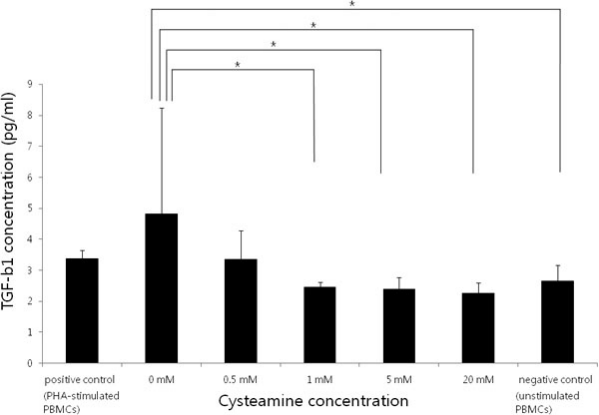
IL-6 levels measured by ELISA. The IL-6 levels decreased with an increase in the CYS concentration (p=0.003; Kruskal–Wallis test). PHA-stimulated PBMCs served as the positive control and unstimulated PBMCs served as the negative control. *: statistically significant by the Mann–Whitney U test.

The TGF-β1 levels decreased with an increase in CYS concentration (p=0.011; Kruskal–Wallis test; Figure 4). A CYS concentration of ≥1 mM significantly lowered the TGF-β1 levels (p=0.034, 0.034, and 0.021, respectively; Mann–Whitney U test).33342.

### PBMC stimulation assay

**Figure 5:**
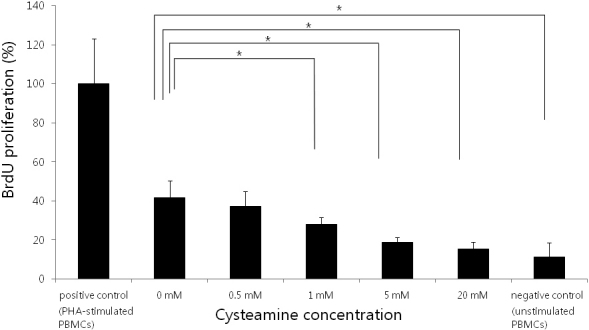
PBMC stimulation test. Cultured human corneal endothelial cells were used as stimulating cells. Cysteamine (CYS) suppressed PBMC proliferation in a dose-dependent manner (p<0.001; Kruskal–Wallis test). PHA-stimulated PBMCs served as the positive control and unstimulated PBMCs served as the negative control. *: statistically significant by the Mann–Whitney U test.

Mitomycin C-treated HCECs were used as stimulating cells. CYS suppressed PBMC proliferation in a dose-dependent manner (p<0.001; Kruskal–Wallis test; Figure 5). PBMC proliferation was suppressed with ≥1 mM of CYS (p=0.016, 0.009, and 0.009, respectively; 

## Discussion

Endothelial rejection involves inflammatory signals, including keratic precipitates and inflammation in the anterior chamber [1,5]. Leukocyte counts in the graft bed are inversely correlated with actuarial graft survival [25]. Corneal endothelial rejection has been known as the most severe form of rejections [26,27]. Two forms of endothelial rejection have been described [27]. Khodadoust line in endothelial rejection usually originates at a vascularized area of the cornea [27], or at the site of an anterior synechiae [26]. Within a few days, the line extends across the donor cornea, destroying endothelial cells, depositing keratic precipitates. The other form is diffuse endotheliitis although diffuse keratic precipitates are not visible. Endothelial rejection is one of inflammatory responses. Endothelial rejection is one of inflammatory responses although the corneal endothelial cell rejection has been known to be mediated by a chronic adaptive immunity [28] and it is not well established that simply inflammation plays a major role in this process. Thus, the interaction between PBMC and HCEC is important for graft survival.

In this study, we showed that CYS suppresses PBMC proliferation and decreases intracellular ROS levels in a dose-dependent manner. CYS is an antioxidant that enhances intracellular GSH levels. Moreover, CYS may act as a scavenger of superoxide free radicals and hydrogen peroxide [29]. ROS have been described to modulate inflammation and tissue repair [30]. Although ROS causes cell death at high level [22], a low level of ROS has been described to mediate cell signaling events in inflammation as a second messenger [30]. Reduction of intracellular ROS can reduce the cell proliferation and inflammatory response. Nuclear factor-erythroid 2-related factor 2 (Nrf2), a redox-sensitive transcription factor, has been implicated in cellular responses to oxidative stress; Nrf2 inhibits nuclear factor kappa B (Nf-κB) [31,32], which plays a key role in immune response and inflammation [33]. Thus, CYS may exert an anti-inflammatory effect by reducing intracellular ROS formation. In addition, CYS suppressed the levels of IL-6 and TGF-β1, which are the cytokines secreted by PBMCs [34,35]. Nf-κB has been reported to induce the activation of IL-6 gene expression [36,37] and to be regulated by TGF-β1 [38,39]. IL-6 is a proinflammatory cytokine that plays multiple roles during injuries and inflammation [32]. IL-6 levels in the human aqueous humor have been reported to rise during corneal endothelial immune reactions [40,41]. It has been reported that the specific release of proinflammatory cytokines from alloreactive infiltrating cells, results in apoptosis in the corneal endothelium [42]. TGF-β1 is a multifunctional cytokine [43]. Although the role of TGF-β1 in corneal endothelial rejection is yet unclear, TGF-β1 has been reported to be induced by tissue injuries, participate in tissue fibrosis [40], and regulate inflammation and fibrosis [44]. TGF-β1 has been reported to modulate Nf-κB [38,39], which plays a key role in inflammatory response [33]. Thus, TGF-β1 might regulate the immune response during corneal endothelial rejection.

In our previous study, we showed that, while 10 mM of CYS is not toxic to cultured corneal endothelial cells, higher concentrations of CYS are lethal [45]. We also reported that CYS protects HCECs against oxidative injury-mediated cell death by inhibiting ROS production at high concentrations of CYS [45]. Therefore, low concentrations of CYS could not only effectively suppress inflammation but also be nontoxic to corneal endothelial cells.

In conclusion, this study showed that CYS decreased PBMC proliferation, IL-6 and TGF-β1 levels via ROS formation. Our results suggest that CYS could suppress inflammation associated with PBMCs to corneal endothelial cells. However, further in vivo studies are required.
